# EMP: Enhanced Multi-modal Prediction for fashion sales using Fourier Mapping and ERP-based contrastive learning

**DOI:** 10.1371/journal.pone.0350107

**Published:** 2026-06-16

**Authors:** Sanguk Park, Byungyoon Park, Seohyun Lee, Subin Shin, Wooju Kim

**Affiliations:** 1 Department of Industrial Engineering, Yonsei University, Seoul, Republic of Korea; 2 Department of Artificial Intelligence, Yonsei University, Seoul, Republic of Korea; University of Dhaka, BANGLADESH

## Abstract

Predicting sales for newly introduced fashion products presents a significant challenge due to the absence of historical data and the inherently multi-modal nature of the inputs. To address this issue, we propose the Enhanced Multi-modal Prediction (EMP) model, a novel framework for zero-shot time series forecasting. EMP follows a two-stage architecture: (1) ERP-Aware Contrastive Learning, which retrieves reference items using Edit Distance with Real Penalty (ERP), a metric-based elastic distance designed to measure similarity between real-valued time series, to construct a meaningful embedding space, and (2) a Transformer-based encoder-decoder, which predicts 12-week sales trajectories using Fourier-mapped embeddings. Unlike prior models that rely on external signals, EMP leverages internal reference item sales, thereby eliminating dependency on third-party APIs and enhancing forecasting accuracy. It introduces Fourier Mapping, which generates scale-aware embeddings that preserve the absolute magnitude of sales values while enabling interpretability through inverse mapping. This representation mitigates spectral bias and allows the model to effectively capture both low- and high-frequency sales patterns. To maximize this benefit, Fourier Mapping is applied selectively to internal reference item sales, where high frequency variation is more prominent than in external signals. We further present a theoretical analysis of the Lipschitz continuity of Fourier Mapping, demonstrating that it maintains a higher Lipschitz constant than conventional scaling methods. This property ensures the preservation of fine-grained input variations, thereby improving the model’s sensitivity to subtle fluctuations in sales trajectories. Experiments on both private and benchmark datasets demonstrate that EMP achieves superior forecasting accuracy compared to existing methods, validating its effectiveness in fashion sales prediction. The implementation of our method is available at the following URL: https://github.com/sanguk-ys/EMP.

## Introduction

Predicting the sales of newly introduced fashion items is a crucial challenge for the retail and fashion industries [1]. Accurate forecasting enables better inventory management, supply chain optimization, and cost reduction while minimizing the risks associated with overstocking or stockouts [2]. However, the complex nature of fashion data, characterized by its multi-modal features such as visual images, textual descriptions, temporal patterns, and contextual signals, poses significant challenges for traditional forecasting methods [3].

Fashion sales forecasting involves capturing intricate relationships between products, temporal patterns, and external trends [4]. Traditional methods, such as statistical models and simple machine learning techniques, often fail to utilize the rich multi-modal information embedded in fashion datasets. Additionally, the lack of sufficient historical data for new products, known as the zero-shot forecasting problem, further complicates the task [5]. To address these limitations, a more sophisticated model capable of integrating multi-modal data and leveraging external signals is required [6].

This paper introduces the Enhanced Multi-modal Prediction (EMP) model, a novel framework designed to predict the 12-week sales quantities of newly launched fashion products. EMP incorporates a two-stage architecture that effectively combines multi-modal inputs with advanced learning techniques:

1. **Stage 1: ERP-Aware Contrastive Learning**: This stage identifies the Top-10 reference items most similar to the target item based on Edit Distance Real Penalty (ERP). Contrastive learning is employed to optimize the embedding space by learning the relationships between anchor, positive, and negative samples.
2. **Stage 2: Time Series Forecasting**: Multi-modal features, combined with Fourier-mapped embeddings of the reference item sales, are passed through a Transformer-based encoder-decoder to predict 12-week sales embeddings.

The contributions of **EMP** are summarized as follows:

1. **ERP-Based Reference Item Sales for Accurate and Internally Grounded Forecasting**: EMP introduces a contextual signal modeling strategy that leverages ERP (Edit Distance with Real Penalty) to retrieve reference items with similar sales patterns using contrastive learning. Unlike previous approaches that rely on external signals such as search engine trends [6] or POP signals [7], EMP performs forecasting using internal signal, reference item sales available within the system. By eliminating the need for third-party APIs that are used to obtain external signals, EMP avoids costs typically incurred when using external APIs in commercial applications while improving forecasting accuracy.
2. **Fourier Mapping for High-Fidelity Sales Representation**: Instead of conventional scaling, EMP employs Fourier Mapping to transform sales values into frequency embeddings. This yields scale-aware representations that preserve absolute magnitudes while also reducing spectral bias. This enables the model to capture high-frequency variations in sales patterns. To support this, we theoretically analyze the Lipschitz continuity of Fourier Mapping and show that it maintains a higher Lipschitz constant than max normalization.
3. **Inverse Fourier Mapping for Scalar Sales Value Reconstruction** EMP applies Inverse Fourier Mapping to convert predicted continuous Fourier embeddings back into discrete scalar sales values. This transformation ensures that the model outputs interpretable numerical predictions while maintaining consistency with the frequency-based learned representation.

Extensive experiments conducted on both private and benchmark datasets demonstrate that EMP achieves state-of-the-art (SOTA) performance, outperforming traditional and recent models, including outperforming various traditional and deep learning-based models, including KNN-based, RNN-based, and Transformer-based architectures. These results highlight the efficacy of ERP-Aware Contrastive Learning and Fourier Mapping in capturing inter-item relationships and complex temporal patterns.

## Related works

### Time series forecasting

Time series forecasting has seen significant advancements with the adoption of deep learning methods. Transformer-based architectures have played a central role in this progress.

Models like Informer [8], Autoformer [9], and Fedformer [10] utilize attention mechanisms to capture long-term dependencies in time series data. Temporal Fusion Transformer (TFT) [11] introduces a flexible encoder-decoder structure for interpretable multi-horizon forecasting, explicitly modeling static covariates and known future inputs. PatchTST [12] reformulates time series as non-overlapping patches, effectively learning local patterns and improving long-term forecasting through patch-wise attention. Non-stationary Transformer [13] addresses distributional shifts by adaptively re-weighting attention using temporal statistics, improving robustness to evolving dynamics. Crossformer [14] emphasizes inter-variable dependencies by modeling cross-dimensional attention across time and features, achieving strong performance on multivariate benchmarks.

Despite these advances, most existing models focus on single-modal or structured numerical inputs, often neglecting the potential of integrating multi-modal information such as visual and textual context.

### Sales forecasting

Sales forecasting for newly introduced fashion products presents distinct challenges, primarily due to the sector’s volatility, rapid product turnover, and limited historical data availability. Traditional forecasting methodologies often fall short in this dynamic environment, necessitating the adoption of advanced techniques that leverage modern data analytics and machine learning.

A prominent approach involves integrating exogenous data sources such as Google Trends into predictive models. [6] demonstrated that combining Google Trends data with visual and metadata features via neural networks significantly enhances forecasting accuracy, particularly in the absence of historical sales data. This multimodal methodology exploits consumer interest signals, a crucial factor for demand prediction in fast-paced markets. Similarly, [15] highlighted the importance of incorporating customer search data into traditional time series models, confirming that external data improves forecasting precision and underscores the necessity of contextual information.

The application of advanced machine learning techniques has further revolutionized sales forecasting. Deep learning models, as emphasized by [16], effectively capture intricate demand patterns and nonlinear relationships inherent in consumer behavior. Ensemble methods, such as XGBoost, have also demonstrated their efficacy. [17] illustrated how combining multiple predictive algorithms enhances forecasting performance in diverse datasets.

Challenges associated with limited data for new products have been addressed through innovative approaches. Siamese neural networks, as proposed by [18], enable comparisons between new products and existing ones by learning relational embeddings based on product attributes. This technique is particularly relevant in the fashion industry, where products frequently enter the market without prior sales history. Additionally, addressing lost sales is critical for retailers, as inventory shortages often distort demand signals. [19] introduced a two-layer model to account for censored demand, enhancing both forecasting accuracy and inventory management.

From this perspective, integrating multimodal data, external signals, and advanced modeling techniques emerges as a critical component for effective sales forecasting of new fashion products.

### ERP-based similarity and contrastive learning

Edit Distance with Real Penalty (ERP) [20] is an elastic distance measure for real-valued time series, closely related to Dynamic Time Warping (DTW) [21–23]. ERP has been further examined in prior studies on time series similarity and classification [24,25]. While DTW effectively handles temporal misalignment, it does not satisfy the triangle inequality and is therefore not a proper metric. In contrast, ERP satisfies the metric properties, including the triangle inequality, which ensures consistent and transitive distance relationships among samples. This property is critical for contrastive learning, as it ensures that the learned embedding space reflects consistent distance relationships.

Contrastive learning [26,27], particularly triplet-based approaches, has emerged as an effective technique for learning discriminative embeddings. By minimizing the distance between anchor-positive pairs while maximizing the distance to negative samples, contrastive learning creates a structured embedding space that captures inter-item relationships. EMP combines ERP and contrastive learning to retrieve reference items that share meaningful temporal and contextual similarities with the target item, thereby enhancing the forecasting pipeline.

### Fourier mapping and spectral bias mitigation

Fourier Transform has been extensively employed in time series forecasting to extract periodic and frequency-based patterns. Models such as Autoformer [9] and FEDformer [10] utilize Fourier Transform to decompose time series data into seasonal and trend components, enhancing their ability to perform efficient long-term forecasting. These approaches focus on transforming the time series into the frequency domain, which is particularly effective for capturing global temporal dependencies.

While these methods operate at the sequence level, another line of research explores the use of Fourier-based transformations at the input level to improve neural network learning dynamics. In particular, recent studies have highlighted a phenomenon known as spectral bias: as shown by Rahaman et al. [28], neural networks tend to learn low-frequency functions more easily than high-frequency ones, making it difficult to capture rapid changes or fine-grained variations in data.

This limitation is partly attributed to the structure of the neural tangent kernel (NTK), whose eigenvalue spectrum decays rapidly, resulting in poor representation of high-frequency components. To mitigate this issue, Tancik et al. [29] introduced Fourier Features, which encode input coordinates into a high-dimensional sinusoidal space. This transformation flattens the NTK spectrum, increasing the network’s sensitivity to high-frequency variations and accelerating convergence for complex signal representations.

In the context of predicting individual sales values, low-frequency components represent the baseline level of the value, reflecting broader and smoother characteristics, while high-frequency components capture finer variations or noise embedded within the value itself. This bias limits the ability of models to fully represent the detailed variations present within each sales value, which are critical for accurate forecasting.

To address this limitation, this study introduces Fourier Mapping, a novel technique that applies frequency-based transformations at the granularity of individual data points instead of the entire time series. Unlike [29]’s Fourier Features, which are designed to encode spatial coordinates (e.g., pixel locations) in applications such as image synthesis, our Fourier Mapping is specifically developed for time series forecasting. Rather than applying frequency-based transformations to coordinate data, Fourier Mapping transforms individual values of time series into high-dimensional frequency embeddings. This allows the model to capture both low-frequency components and high-frequency components of sales.

Furthermore, this study introduces Inverse Fourier Mapping, a mechanism that allows the model to convert predicted Fourier embeddings back into discrete sales values. While Fourier Features serve as a static transformation without a direct inverse mapping, Fourier Mapping ensures interpretability by enabling the reconstruction of numerical outputs from frequency-based representations.

A key advantage of Fourier Mapping is its ability to preserve the original scale of sales values, which is often lost during preprocessing in traditional forecasting models. Prior approaches, such as “Well Googled is Half Done” [6] and POP Signal [7], apply max normalization to rescale sales data before feeding it into the model. While this technique ensures comparability across products and facilitates learning relative differences, it also removes scale-related information that may be crucial for capturing absolute sales magnitudes. In contrast, Fourier Mapping maintains the original scale during transformation, allowing the model to leverage both relative variations and absolute magnitudes for more informative representation.

### Limitations of normalization in time series forecasting

Existing Time Series Models typically rely on dataset-specific normalization techniques to preprocess numerical values before inputting them into deep learning architectures. Models such as Informer [8], Autoformer [9], and FEDformer [10] use either min-max normalization or standardization, defined as:


$$\begin{array}{c} x^{\prime }=\frac{x-\min(x)}{\max(x)-\min(x)},\;x^{\prime }=\frac{x-\mu }{\sigma } \end{array}$$ (1)

While these methods allow models to operate within a fixed range, they suffer from the following limitations:

- **Dataset Dependency:** The normalization parameters (min(x),max(x),μ,σ) are computed per dataset, making it difficult to generalize across datasets with varying sales distributions.
- **Scaling Mismatch:** When applying a pre-trained model to new datasets, the rescaled values may differ significantly, leading to distribution shifts.
- **Sensitivity to Outliers:** Extreme values can distort the normalization process, reducing the meaningfulness of relative differences between numerical values.

To address these limitations, we introduce Fourier Mapping as a normalization-free transformation. Unlike dataset-specific normalization methods, Fourier Mapping encodes raw numerical values directly into frequency-based embeddings, preserving their original scale without requiring dataset-dependent rescaling. This design enables consistent numerical representation across datasets, which can support better transferability by eliminating the need for dataset-specific scaling.

## Problem definition

### Overall problem formulation

Given a dataset of fashion products i∈{1,…,N}, the objective is to predict the sales quantities over a 12-week horizon (*H* = 12). Each product is associated with a set of multi-modal features:

- **Image Features** ($F_{img}\in {\mathbb{R}}^{d_{img}}$): Encoded representations of the product’s visual attributes, extracted using a pre-trained CLIP model [30].
- **Text Features** ($F_{text}\in {\mathbb{R}}^{d_{text}}$): Embeddings derived from product descriptions, obtained via a pre-trained CLIP model [30].
- **Temporal Features** ($F_{temp}\in {\mathbb{R}}^{3}$): Scalar values representing the release date, including week, month, and year, which are processed separately as numerical inputs.
- **Metadata** ($F_{meta}\in {\mathbb{R}}^{d_{meta}}$): One-hot encoded categorical information, such as product category, fabric, color, and brand, directly input into the model.
- **Reference Item Sales Data** ($S_{k-item}\in {\mathbb{R}}^{N_{ref}\times H}$): Sales values of *N*_*ref*_ reference items over *H* weeks, flattened into a single vector of size ${\mathbb{R}}^{N_{ref}\times H}$ and transformed using the Fourier Mapping function $\gamma (S_{k-item})$, where $N_{ref}=10$ represents the number of reference items selected for each target item.

The Fourier Mapping function γ(s) transforms a scalar sales value *s* into a high-dimensional frequency embedding:


$$\begin{array}{c} \gamma (s)=\left[\sin(f_{1}s),\cos(f_{1}s),\ldots ,\sin(f_{d/2}s),\cos(f_{d/2}s)\right], \end{array}$$ (2)

where:

- $f_{k}=\exp\left(-\frac{\log(B)}{d}\cdot 2(k-1)\right),\;k\in \left\{1,2,\ldots ,\frac{d}{2}\right\}$,
- *d* is the embedding dimension,
- *s* is the scalar sales value, and
- *B* is a scaling factor that controls the frequency range of the Fourier basis.

The proposed model learns a mapping function:


$$\begin{array}{c} 𝐟:\left(\begin{array}{c} F_{img},F_{text},F_{temp},F_{meta},\gamma (S_{k-item}) \end{array}\right)\to {\hat{𝐅}}_{sales}^{fourier}, \end{array}$$ (3)

where ${\hat{𝐅}}_{sales}^{fourier}=[{\hat{e}}^{1},{\hat{e}}^{2},\ldots ,{\hat{e}}^{12}]$ represents the predicted Fourier-mapped embeddings for sales over the 12-week horizon.

To obtain the final sales prediction $\hat{S}$ in scalar form, Inverse Fourier Mapping is performed. This allows the reconstructed values to be compared with actual sales values.

### Stage 1: ERP-aware contrastive learning

In Stage 1, the model retrieves the Top-10 reference items using ERP distance. The multi-modal input features are fused into an embedding vector **E**_*i*_ via:


$$\begin{array}{c} {𝐄}_{i}={𝐟}_{ECL}(F_{img},F_{text},F_{temp},F_{meta}), \end{array}$$ (4)

where **f**_*ECL*_ is a function representing ERP-Aware Contrastive Learning.

Here, *i* represents the anchor item, and *j* represents the candidate reference items:


$$\begin{array}{c} D_{i,j}=\text{Euclidean distance}({𝐄}_{i},{𝐄}_{j}), \end{array}$$ (5)

where **E**_*i*_ and **E**_*j*_ are the embeddings for the anchor item *i* and candidate reference item *j*, respectively.

The nearest reference items are selected as:


$$\begin{array}{c} {𝐏}_{i}=\text{Argsort}(D_{i,1},D_{i,2},...,D_{i,N})[:N_{ref}], \end{array}$$ (6)

where the function Argsort returns the indices of candidate items sorted in ascending order of Euclidean distance. The *P*_*i*_ serve as reference items in Stage 2.

### Stage 2: Time series forecasting

In Stage 2, a Transformer-based encoder-decoder is employed to predict the 12-week Fourier-mapped sales embeddings. The inputs to the decoder include both multi-modal features and the Fourier embeddings of reference item sales:


$$\begin{array}{c} {\hat{𝐅}}_{sales}^{fourier}={𝐟}_{TSF}(F_{img},F_{text},F_{temp},F_{meta},\gamma (S_{k-item})) \end{array}$$ (7)

where **f**_*TSF*_ represents the Transformer-based encoder-decoder architecture.

## Model architecture

EMP integrates a Transformer encoder-decoder structure with the following components:

- **ERP-Aware Contrastive Learning for Reference Retrieval:** Retrieves reference items most similar to the target item using ERP distance.
- **Reference Item Sales Encoding:** Encodes sales patterns of reference items selected based on ERP distance using Fourier Mapping and structured masking.
- **Feature Fusion Network and Decoder:** Fuses image, text, temporal, and metadata features to generate robust embeddings.
- **Fourier Mapping and Inverse Mapping:** Converts sales values to frequency embeddings and back to scalar values.

**Fig 1:**
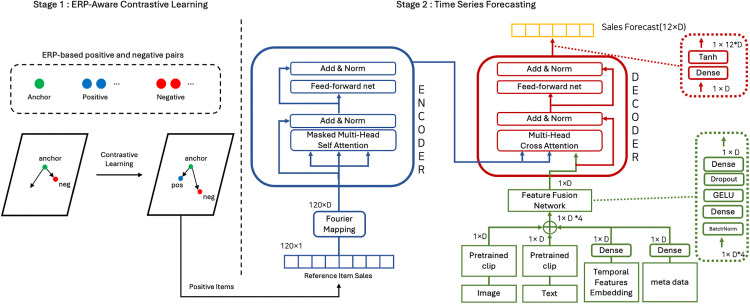
EMP Model Architecture. Note. Stage 1 (ERP-Aware Contrastive Learning) retrieves top-*K* positive reference items for a given target item by measuring sales similarity using ERP distance. These selected items serve as reference item sales in Stage 2 (Time Series Forecasting). In Stage 2, the model encodes multi-modal features (image, text, metadata, temporal) alongside Fourier-mapped sales embeddings of the reference items using a Transformer-based encoder-decoder. The predicted output is a 12-week sales forecast represented as Fourier embeddings.

The proposed model architecture is shown in Fig 1.

### ERP-Aware contrastive learning

ERP-Aware Contrastive Learning is a framework designed to effectively learn complex inter-item relationships in time series forecasting. This approach utilizes ERP (Edit Distance with Real Penalty) Distance to define relationships among anchor, positive, and negative items, optimizing inter-item distances through contrastive loss to establish a clear embedding space.

The given dataset comprises Temporal Features, Image Features, Text Features, and Metadata. Temporal Feature Encoders learn temporal patterns, Image Encoders extract visual characteristics from product images, Text Encoders analyze contextual information from descriptions and reviews, and Metadata Encoders capture categorical attributes such as the category, color, fabric, and brand of the target item. These features are fused into a unified embedding vector:


$$\begin{array}{c} 𝐅=\text{LayerNorm}([{𝐅}_{temp},{𝐅}_{img},{𝐅}_{text},{𝐅}_{meta}]) \end{array}$$ (8)

where **F** represents the unified embedding vector, and **F**_*temp*_, **F**_*img*_, **F**_*text*_, **F**_*meta*_ correspond to temporal, image, text, and metadata embeddings.

Using the ERP Distance matrix, the distance between item sales time series is calculated. Positive items are defined as the top 10 closest items based on ERP Distance, while negative items are those farther away. This is expressed as:


$$\begin{array}{c} \text{Positive\_Items}(i)=\text{Argsort}(\text{ERP Distance Matrix}(i))[:N_{ref}] \end{array}$$ (9)

$$\begin{array}{c} \text{Negative\_Items}(i)=\text{Argsort}(\text{ERP Distance Matrix}(i))[N_{ref}:] \end{array}$$ (10)

Here, Positive items are the nearest, and Negative items are the farthest based on ERP Distance.

**ERP Distance:** ERP quantifies the similarity between two time series by incorporating penalties for insertions, deletions, and substitutions of continuous values. For two time series *X* and *Y*, it is recursively defined as:


$$\begin{array}{c} \text{ERP}(X,Y)=\min\{\begin{array}{c} \text{ERP}(X_{1:m-1},Y_{1:n-1})+d(x_{m},y_{n}),\\ \text{ERP}(X_{1:m-1},Y)+d(x_{m},g),\\ \text{ERP}(X,Y_{1:n-1})+d(g,y_{n}) \end{array}\} \end{array}$$ (11)

where:

- *x*_*m*_ and *y*_*n*_ are the last elements of *X* and *Y*,
- *g* is the reference value (commonly 0),
- *d*(*x*, *y*) is the Euclidean distance, $d(x,y)=(x-y{)}^{2}$.

ERP considers the temporal order while matching patterns between time series and accounts for penalties for insertions and deletions based on continuous values.

**Hard Negative Mining:** The hardest positive and negative items are critical in the learning process. The hardest positive is the farthest among the positive items, and the hardest negative is the closest among the negative items:


$$\begin{array}{c} D_{pos}=\max(D(a,p_{1}),D(a,p_{2}),\ldots ,D(a,p_{k})) \end{array}$$ (12)

$$\begin{array}{c} D_{neg}=\min(D(a,n_{1}),D(a,n_{2}),\ldots ,D(a,n_{m})) \end{array}$$ (13)

where *D*(*a*,*p*) and *D*(*a*,*n*) are the Euclidean distances between the anchor and positive or negative items, respectively. Hardest positive and negative items are optimized using Triplet Loss:


$$\begin{array}{c} {ℒ}_{ECL}=\max(D_{pos}-D_{neg}+\alpha ,0) \end{array}$$ (14)

Here, α is a margin value ensuring a clear separation between positive and negative items.

Contrastive Loss minimizes the distance between anchor-positive pairs and maximizes the distance between anchor-negative pairs. Two variations of the Triplet Loss, namely MaxPlus Margin Triplet Loss and SoftPlus Margin Triplet Loss, are commonly used. Based on experimental results, the MaxPlus Margin Triplet Loss demonstrated consistently better performance and was thus adopted for this study. The two variations are defined as:


$$\begin{array}{c} {ℒ}_{triplet}=\max(D_{pos}-D_{neg}+\alpha ,0) \end{array}$$ (15)

$$\begin{array}{c} {ℒ}_{triplet}=\log(\exp(D_{pos}-D_{neg})+1) \end{array}$$ (16)

**Dynamic Sampling** ERP-Aware Contrastive Learning employs Sampler for dynamic sampling of positive and negative items. Based on ERP Distance, positive items are the top 10 similar items, and negative items are randomly chosen from the rest. Each training group comprises one anchor, 10 positives, and the remaining negatives. This strategy enhances the network’s ability to learn diverse relationship patterns and ensures a balanced representation of positive and negative samples during training.

ERP-Aware Contrastive Learning quantifies inter-item relationships, distinguishing positive and negative items using ERP Distance and optimizing their embeddings via Triplet Loss. This approach enhances the network’s ability to capture complex patterns and relationships, even in imbalanced or sparse data scenarios, significantly improving prediction accuracy and generalization performance.

### Reference item sales encoding

The encoder architecture in EMP is inspired by [6], which originally used external signals such as Google Trends as input. Similarly, [7] has incorporated POP signal as contextual signal. In contrast, EMP replaces these external signals with reference item sales selected based on ERP distance, utilizing only internal signals.

Given a target item, the top-*K* most similar items are retrieved based on ERP distance. Each of these *K* reference items provides a sequence of *H* historical sales values, which are transformed into frequency embeddings using Fourier Mapping. These *K* × *H* embeddings form the input to a Transformer Encoder, which captures intra-series and inter-series patterns.

**Fig 2:**
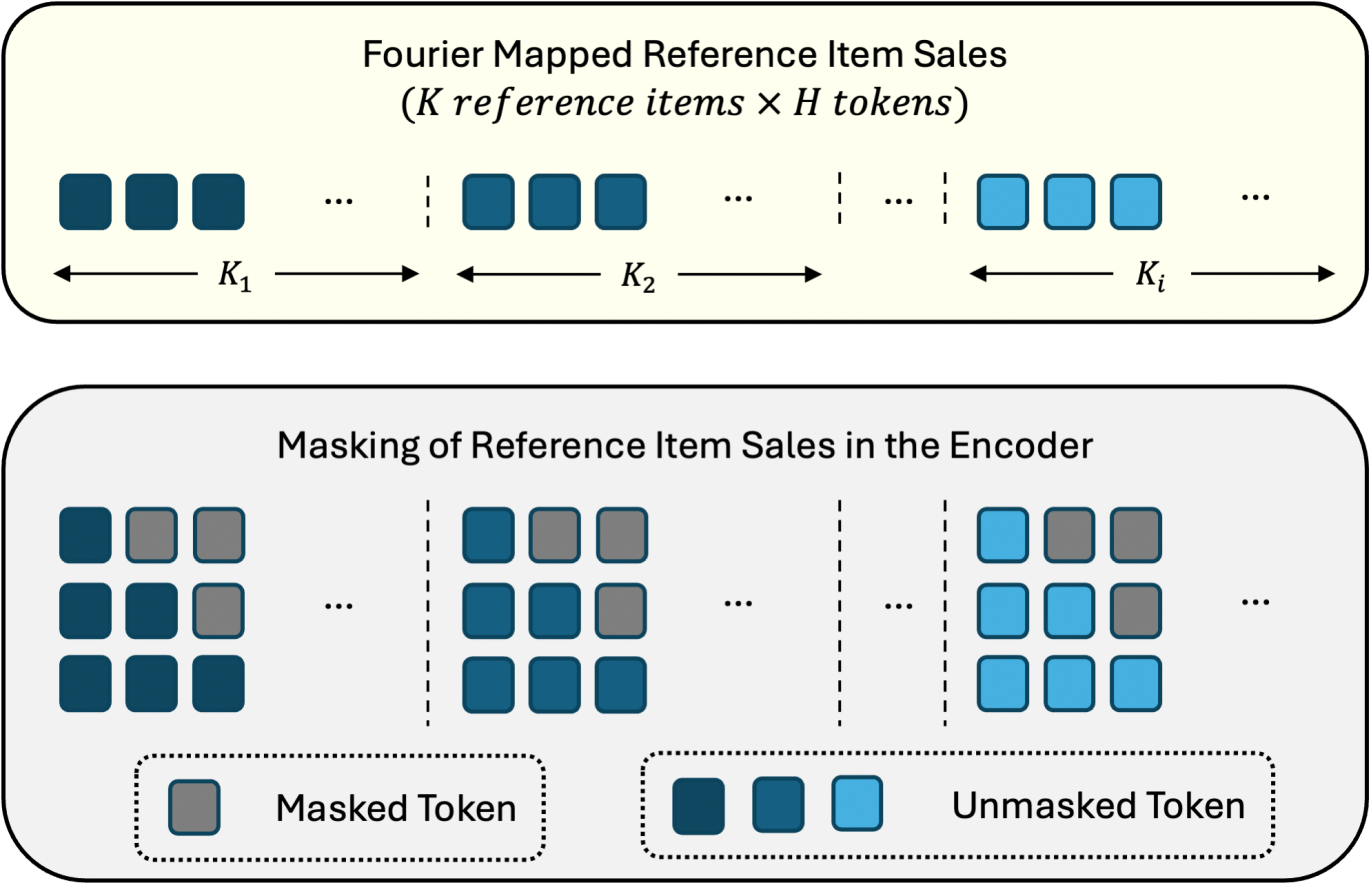
Structured masking of Reference Item Sales in the Encoder. Note. The input consists of *K* reference items, each with *H* weeks of sales, forming *K* × *H* tokens. Masking ensures that each reference item is treated as an independent time series, preventing unintended temporal correlations.

To effectively model inter-series dependencies while preserving causal relationships, a specialized masking mechanism inspired by TIMER-XL [31] is applied to Reference Item Sales (Fig 2). The masking strategy treats each reference item as an independent time series while ensuring structured alignment, allowing the model to process them consistently. A causal mask prevents information leakage from future time steps, enforcing temporal consistency across all reference items. Additionally, cross-item masking regulates interactions between different reference items, preventing excessive dependencies and ensuring that meaningful patterns are learned while maintaining inter-series independence. This structured masking approach enables the model to extract robust dependencies across multiple neighbors while avoiding over-reliance on any single reference item.

### Feature fusion and decoder

The Feature Fusion Network is based on the GTM Transformer [6] architecture and combines image, text, metadata, and temporal features into a single unified representation. Specifically, the modality-specific embeddings are concatenated and normalized using Batch Normalization. The fused representation is then passed through a multi-layer perceptron (MLP) consisting of two linear layers with intermediate Layer Normalization, GELU activation, and dropout, producing the final fused embedding. An overview is provided in Fig 1.

The Decoder is based on the Transformer architecture and takes input from the Contextual Data Encoder and the Feature Fusion Network. The output from the Contextual Data Encoder acts as the Key (*K*) and Value (*V*) in the cross-attention mechanism, while the Query (*Q*) are derived from the output of the Feature Fusion Network. This mechanism enables the Decoder to capture interactions between the contextual information and the integrated features. After each attention step, a Feed-Forward Network (FFN) applies non-linear transformations.

### Fourier mapping

In time series forecasting, effectively learning the complex periodic patterns and high-frequency components of individual sales values is crucial. However, traditional multilayer perceptron (MLP) and Transformer-based models often suffer from spectral bias, focusing primarily on low-frequency components. This limitation hinders their ability to learn detailed changes in sales data, leading to suboptimal predictive performance. To address this, the EMP model employs Fourier Mapping, which captures the frequency characteristics of individual scalar sales values.

The Fourier Mapping function transforms a scalar sales value *s* into a high-dimensional frequency embedding using sine and cosine functions. This embedding is defined across multiple frequency scales, enabling the model to capture both low- and high-frequency patterns effectively. By encoding sales data in this manner, Fourier Mapping mitigates spectral bias and preserves the original scale of the sales values, providing richer feature representations for downstream forecasting tasks.

In the architecture of the EMP model, Fourier Mapping plays a critical role in two key stages:

1. Reference Item Sales Embedding: Fourier Mapping is applied to the 12-week sales data of the top-10 reference items selected through ERP distance. Each of these 10 reference items’ sales time series is transformed into a frequency embedding, which serves as a crucial input to the Encoder. This step ensures that the model can incorporate the sales data in a scaling-free manner, preserving the inherent scale of the sales values and providing the model with richer contextual information about the reference items’ sales dynamics, including high-frequency variations from similar items.
2. Target Embedding for Decoder Output: After the Decoder generates the predicted Fourier embeddings for the target item’s 12-week sales, these embeddings are compared against the ground-truth target embeddings obtained through Fourier Mapping. This comparison acts as the supervisory signal for training, aligning the model’s predictions with the true sales patterns of the target item.

By integrating Fourier Mapping at both the input (reference item embeddings) and output (target embeddings) stages, the EMP model ensures that frequency-based characteristics are effectively leveraged throughout the forecasting pipeline. This dual application enhances the model’s accuracy and its ability to generalize across diverse datasets.

### Inverse Fourier mapping

The final predictions of the model are represented as frequency-based Fourier embeddings. Since the forecasting task ultimately requires scalar sales values, Inverse Fourier Mapping serves as a post-processing step to transform these continuous embeddings into discrete numerical outputs.

**Process of Inverse Fourier Mapping** Given a predicted Fourier embedding vector **E**_*pred*_, Inverse Fourier Mapping reconstructs the corresponding scalar sales value $\hat{s}$ by identifying the most similar scalar representation from the learned Fourier basis vectors. Since the predicted Fourier embeddings exist in a **continuous** frequency space, but sales values are inherently **discrete**, this transformation bridges the gap between these two representations. The reconstruction process is formulated as:


$$\begin{array}{c} \hat{s}=\text{arg}\max({𝐅}^{T}\cdot {𝐄}_{pred}), \end{array}$$ (17)

where:

- **F**: A set of precomputed Fourier basis vectors generated through Fourier Mapping,
- **E**_*pred*_: The predicted Fourier embedding vector output by the model.

As a post-processing step, Inverse Fourier Mapping ensures that the model’s continuous frequency-based outputs are converted into interpretable, discrete sales values. This transformation maintains the learned frequency-based representation while aligning with the discrete nature of real-world sales data.

### Loss function

The learning process of the EMP model is divided into two stages, each optimized with a loss function designed to achieve distinct objectives. The first stage employs ERP-Aware Contrastive Loss to learn relationships among items by optimizing the distances between anchor, positive, and negative samples.

In the second stage, the model predicts Fourier-mapped embeddings of the target sales data. The loss function for this stage minimizes the Mean Squared Error (MSE) between the predicted Fourier embeddings and the ground-truth Fourier embeddings. After training, the predicted Fourier embeddings are inverse-mapped into scalar sales values, which are then used to calculate evaluation metrics.

**Stage 1: ERP-Aware Contrastive Loss** ERP-Aware Contrastive Learning plays a key role in the first stage. ERP (Edit Distance with Real Penalty) quantifies the similarity of temporal patterns, helping distinguish relationships between target and reference items. The loss function for this stage minimizes the distance between positive pairs while maximizing the distance between negative pairs, defined as:


$$\begin{array}{c} {ℒ}_{ECL}=\max(D_{a,p}-D_{a,n}+\alpha ,0) \end{array}$$ (18)

where:

- *D*_*a*,*p*_: Maximum distance in the embedding space between the anchor and positive items
- *D*_*a*,*n*_: Minimum distance in the embedding space between the anchor and negative items in the embedding space
- α: Margin value ensuring a minimum gap between positive and negative items

This loss function encourages minimizing distances to positive items while maximizing distances to negative items, optimizing the embedding space based on ERP Distance.

**Stage 2: Time Series Forecasting Loss** In the second stage, Time Series Forecasting Loss is used to predict future sales quantities based on Fourier-mapped embeddings. Fourier Mapping transforms sales scalar data into frequency-domain embeddings, enhancing the representation of temporal patterns. The loss function minimizes the Mean Squared Error (MSE) between the predicted and true Fourier-mapped embeddings:


$$\begin{array}{c} {ℒ}_{TSF}=\frac{1}{H}\sum_{i=1}^{H}\|{𝐄}_{true,i}-{\hat{𝐄}}_{pred,i}{\|}^{2} \end{array}$$ (19)

where:

- *H*: Number of prediction steps
- **E**_*true*,*i*_: True Fourier-mapped embedding vector
- ${\hat{𝐄}}_{pred,i}$: Predicted Fourier-mapped embedding vector

This loss function ensures that the network accurately predicts Fourier-mapped embeddings, balancing low- and high-frequency components.

### Euclidean distance properties of fourier mapping

A key property of Fourier Mapping, as defined earlier Eq (2), is that the Euclidean distance between two Fourier embeddings depends solely on the absolute difference between the original sales values. Specifically, for two sales values *s*_1_ and *s*_2_ with a difference of $K=|s_{1}-s_{2}|$, their Fourier embeddings exhibit a fixed Euclidean distance


$$\begin{array}{c} D(\gamma (s_{1}),\gamma (s_{2}))=\sqrt{\sum_{k=1}^{2/d}2(1-\cos(f_{k}K))} \end{array}$$ (20)

**Fig 3:**
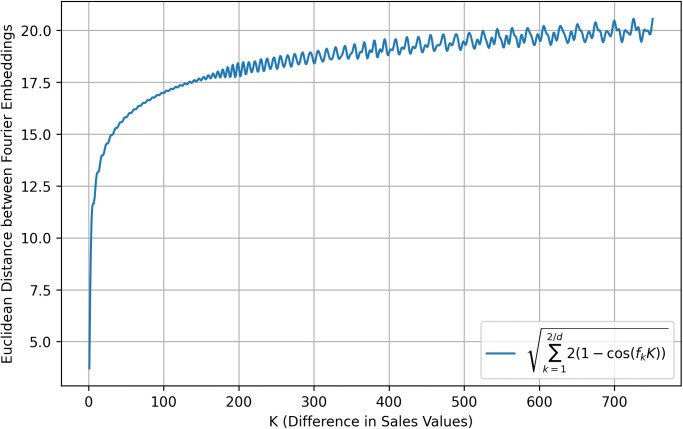
Effect of *K* on Euclidean Distance in Fourier Embeddings.

This function indicates that the Euclidean distance is entirely determined by $K=|s_{1}-s_{2}|$ and independent of the specific values of *s*_1_ or *s*_2_. However, due to the periodic nature of the cosine function, the growth of $D(\gamma (s_{1}),\gamma (s_{2}))$ is not strictly linear with respect to *K*. As shown in Fig 3, the Euclidean distance generally increases with *K*, but exhibits local oscillations arising from the Fourier basis. Despite these oscillations, the overall trend remains upward, meaning that larger differences in sales values still correspond to greater distances on average—making Fourier Mapping suitable for use with regression-based loss functions.

## Dataset

The EMP model was evaluated using real sales data from TBH Global, a fashion company with over 1,800 offline stores and an online presence. TBH Global owns its brands and provided a dataset of approximately 19,000 items from 2013 to 2024. Each item is associated with multi-modal information, such as images and text descriptions. Additionally, the model’s performance was tested on the benchmark dataset Visuelle 2.0 [32]. The following subsections describe the composition of the TBH dataset and its utilization in the model.

### Image data

Each item is represented by a c-cut RGB image, where the garment is laid flat without being worn. The images feature a white background, showing only the front of the garment.

### Text data

Text descriptions, written in Korean, were provided by the MD during the planning stage of the item. Unlike the concatenated tags (color, category) in Visuelle 2.0, these descriptions are detailed sentences, offering richer information about the items.

### Meta data

Meta data includes attributes such as category, fabric, color, and brand, which are one-hot encoded for input.

### Sales data

Sales data is preprocessed at a weekly granularity for 12 weeks. This is the target data that the model aims to predict.

### Temporal data

Temporal data includes the release date of each product. This information is derived from the first sale date and is provided as integers representing year, month, and day.

### Naver trends data

Following the methodology of the paper “Well Googled is Half Done,” [6] Naver search term trends were collected for each item’s category, fabric, and color using the Naver Trends API. For each item, three time series (category trend, fabric trend, and color trend) were obtained, covering 52 weeks prior to the product’s release. Values returned by the API are max-normalized, representing the ratio to the maximum value during the 52-week period.

### POP signal

Using the methodology of the “POP” paper [7], POP signals were generated by concatenating each item’s color and category into a textual query, which was then expanded with the keywords “fashionable” and “unfashionable.” Images were searched for each query over the time interval [t−k−W,t−k], where k=1…52 and *W* is a temporal window set to 4. The top 20 images returned by Google Image Search were collected. Noise images were removed using confident learning, and the cleaned fashionable images were used to extract the POP signal $S(t)=\{s(t-K_{past}),\ldots ,s(t-k),\ldots ,s(t-1)\}$.

The extracted feature $\theta^{\prime }(z)$ was computed using a pre-trained CLIP model [30], trained on a large dataset of fashion images, instead of the ResNet-50 [33] backbone used in the “Pop” paper. The signal value *s*(*t*−*k*) is calculated as the mean cosine similarity between each fashionable image $x_{i}^{\prime }(t-k)$ and the probe image *z* among the $M^{\prime }(t-k)$ downloaded images.

## Results

### Experimental setup

The experimental setup for the EMP model reflects its two-stage architecture, consisting of ERP-aware contrastive learning and time series forecasting with Fourier-based representations.

- **Stage 1:** ERP-aware contrastive learning is used to optimize the item embedding space. Based on ERP distance, the top 10 most similar items to a target item are treated as positive samples, while the remaining items in the group are treated as negative samples. The model is trained using a margin-based contrastive loss with margin α=1. A batch size of 2048 is used for training, enabling efficient construction of large contrastive groups.
- **Stage 2:** Time series forecasting is performed by predicting Fourier-mapped representations of the 12-week sales sequence. The model adopts a Transformer-based encoder-decoder architecture. Both the encoder and decoder use a hidden dimension of 512. The encoder consists of 2 layers with 4 attention heads, while the decoder consists of 2 layers with 8 attention heads. A batch size of 128 is used for training. The number of ERP-based nearest neighbors is set to 10.

Both stages are trained using the Adam optimizer with a learning rate of 1 × 10^−4^. In addition, a ReduceLROnPlateau scheduler is applied only in Stage 2, with a factor of 0.5 and a patience of 5. Dropout with a rate of 0.2 is applied for regularization. The embedding dimension, including Fourier embeddings, is set to 512. The Fourier mapping scaling factor is set to *B* = 10000, and the forecasting horizon is 12 weeks.

All experiments are conducted in a consistent hardware and software environment, and a fixed random seed was used to ensure reproducibility.

### Datasets

The experiments utilized the TBH Global Dataset and the Visuelle 2.0 Dataset:

- **TBH Global Dataset:** This dataset contains approximately 19,000 fashion products, including images, text descriptions, weekly sales data, and time series data based on Naver Trends. The dataset spans sales records from 2013 to 2024, with the prediction goal being the sales quantities for the next 12 weeks.
- **Visuelle 2.0 Dataset:** This dataset comprises approximately 5,355 products from the Nuna Lie brand, including product images, text tags, weekly sales data, and external time series data based on Google Trends. It is recorded at a weekly granularity and was curated for training and validating the EMP model.

Both datasets include multi-modal characteristics, making them suitable for validating the EMP model’s performance across various modalities.

### Metrics

The performance of the EMP model was evaluated using three metrics:

1. **Mean Absolute Error (MAE):**
  $$\begin{array}{c} \text{MAE}=\frac{1}{H}\sum_{i=1}^{H}|y_{i}-{\hat{y}}_{i}| \end{array}$$ (21)

MAE measures the average absolute error between the predicted (${\hat{y}}_{i}$) and actual (*y*_*i*_) values, providing an intuitive representation of the model’s prediction error.

2. **Weighted Absolute Percentage Error (WAPE):**
  $$\begin{array}{c} \text{WAPE}=\frac{\sum_{i=1}^{H}|y_{i}-{\hat{y}}_{i}|}{\sum_{i=1}^{H}y_{i}} \end{array}$$ (22)

WAPE measures the prediction error relative to the total sales, providing a normalized evaluation of forecasting accuracy [34].

3. **Adjusted Symmetric Mean Absolute Percentage Error (adj-SMAPE):**
  $$\begin{array}{c} \text{adj-SMAPE}=\frac{1}{H}\sum_{i=1}^{H}\frac{\left|y_{i}-{\hat{y}}_{i}\right|}{(|y_{i}|+|{\hat{y}}_{i}|)/2} \end{array}$$ (23)

adj-SMAPE normalizes relative prediction errors, effectively reducing the impact of outliers [35].

These metrics provide comprehensive evaluations of the model’s predictive accuracy.

### Main results

The EMP model outperformed KNN-based models (Attribute KNN, Embedding KNN, Attr+Emb KNN) [36], RNN-based models (Image RNN, Multi-Modal RNN, Cross-Attention RNN) [36], and Transformer-based model (GTM-Transformer, POP) [6,7] on both the TBH Global Dataset and the Visuelle 2.0 Dataset. The input modalities used in the evaluation are as follows: N (Naver Trends), G (Google Trends), I (Image), T (Text), P (POP Signals), R (Reference Item Sales). The results are summarized in the table below(Table 1, 2).

Across both datasets, EMP consistently outperformed all baseline models across all evaluation metrics. This improvement is attributed to the effective learning of inter-item similarities through ERP-Aware Contrastive Learning and the precise capture of high-frequency sales patterns via Fourier Mapping.

**Table 1:** Comparison of EMP and baseline models (TBH Global).

Model	Inputs	M ↓	S ↓	W ↓
Attribute KNN	N	34.471	0.652	0.931
Embedding KNN	I	41.816	0.544	1.133
Attr+Emb KNN	NI	42.438	0.576	1.148
Image RNN	NI	42.093	0.547	1.144
Multi-Modal RNN	NIT	41.471	0.576	1.127
Cross-Attention RNN	NIT	32.748	0.489	0.890
GTM-Transformer	NIT	36.332	0.513	1.101
POP	PIT	35.935	0.510	1.084
EMP (Proposed)	RIT	**29.089**	**0.445**	**0.772**

Note. M: Mean Absolute Error, S: Adjusted Symmetric Mean Absolute Percentage Error, W: Weighted Absolute Percentage Error.

**Table 2:** Comparison of EMP and baseline models (Visuelle 2.0).

Model	Inputs	M ↓	S ↓	W ↓
Attribute KNN	G	18.862	0.583	1.150
Embedding KNN	I	22.718	0.618	1.389
Attr+Emb KNN	GI	23.261	0.615	1.420
Image RNN	GI	18.356	0.591	1.095
Multi-Modal RNN	GIT	19.039	0.651	1.136
Cross-Attention RNN	GIT	17.753	0.585	1.059
GTM-Transformer	GIT	19.937	0.599	1.189
POP	PIT	20.677	0.600	1.233
EMP (Proposed)	RIT	**15.246**	**0.564**	**0.909**

### Comparison with recent time-series forecasting models

To provide additional context against recent state-of-the-art time-series forecasting architectures, we further compare EMP with Informer [8], Autoformer [9], FEDformer [10], and PatchTST [12]. These models operate exclusively on numerical time-series features and do not support multimodal inputs such as images or text. Therefore, to ensure a fair and compatible evaluation protocol, all models – including EMP – were evaluated in a unimodal setting using only the reference-item sales sequence as input. The results on the Visuelle 2.0 dataset are reported in Table 3.

**Table 3:** Comparison of EMP and recent time-series forecasting models on Visuelle 2.0.

Model	M ↓	S ↓	W ↓
EMP (w/o Multi-modal Data)	**15.372**	**0.573**	**0.916**
Informer	23.313	0.741	1.390
Autoformer	31.334	0.715	1.868
FEDformer	28.676	0.711	1.710
PatchTST	20.819	0.607	1.241

Across all metrics, EMP (w/o Multi-modal Data) achieves the strongest performance, despite all models being evaluated under the same 10-variate reference-item input. This result reflects a structural distinction between EMP and recent Transformer-based forecasting architectures, which are designed for classical long-horizon forecasting, where a continuous time series is extrapolated into the future. Their inductive biases rely on assumptions such as temporal continuity between past and future segments—an assumption that does not hold in the reference- driven setting considered here, where the input sequences originate from different items and therefore do not form a single coherent trajectory. This mismatch is especially pronounced for architectures such as Autoformer and FEDformer, whose frequency-based designs assume that historical and future values share consistent spectral structures. In our setting, however, the available sales sequences are short (length 12), providing insufficient temporal resolution for meaningful spectral decomposition. Moreover, because the reference item and target item series do not exhibit aligned frequency characteristics, these models are inherently less suited to this cross-item forecasting scenario, which is reflected in their lower accuracy compared with the other models.

### Ablation study

#### Ablation study on Key EMP modules.

To isolate the contribution of EMP’s core architectural components, we design ablations that disable exactly one module at a time while keeping all remaining parts of the model unchanged. Three components are considered essential: (i) multimodal fusion, (ii) ERP-Aware Contrastive Learning, and (iii) Fourier Mapping, including both the forward transformation and the inverse Fourier reconstruction. Because EMP performs fusion through feature-level integration rather than a dedicated fusion block, ablating multimodal fusion is implemented by removing all multimodal inputs (image, text, metadata, and temporal features). This is the correct operational analogue for testing whether heterogeneous modalities offer complementary information beyond internal reference-item sales. For ERP-Aware Contrastive Learning, ablation is conducted by disabling the contrastive stage entirely. Without the ERP-structured embedding space, reference items cannot be retrieved based on similarity; instead, they are sampled at random from the remaining items. This directly evaluates whether ERP-guided retrieval is necessary for supplying meaningful contextual signals to the forecasting module. For Fourier Mapping, ablation replaces the Fourier embeddings with max-normalized scalar values. This simultaneously removes the forward Fourier transformation applied to reference-item sales and the inverse Fourier mapping used to reconstruct scalar predictions. Thus, this variant evaluates the impact of removing the entire frequency-domain representation mechanism.

The results in Table 4 show that removing either multimodal inputs or ERP-Aware Contrastive Learning results in moderate but consistent performance degradation, indicating that the model benefits from both heterogeneous feature cues and structured reference retrieval. The largest decline is observed when Fourier Mapping is replaced with max-normalized scalar inputs, demonstrating that frequency-based representations—and their inverse reconstruction—play a crucial role in preserving scale information and capturing high-frequency variation.

**Table 4:** Ablation Study on Key EMP Modules on TBH Global and Visuelle 2.0.

Model Variation	Visuelle 2.0			TBH Global		
	M ↓	S ↓	W ↓	M ↓	S ↓	W ↓
Full EMP Model	15.246	0.564	0.909	29.089	0.445	0.772
w/o Multi-modal Data	15.372	0.573	0.916	30.486	0.480	0.809
w/o ERP Contrastive Learning	15.408	0.574	0.918	29.090	0.451	0.774
w/o Fourier Mapping	20.059	0.597	1.196	35.921	0.510	1.083

#### Ablation study on mapping and contextual signal type.

We evaluate the impact of the numerical mapping method and the choice of contextual signal on forecasting performance. As shown in Table 5, we compare Max Normalization and Fourier Mapping across three contextual signals: Google Trends, POP signals, and Reference Item Sales. The evaluation was conducted on both the Visuelle 2.0 and TBH Global datasets using three error metrics.

**Table 5:** Comparison of Max Normalization and Fourier Mapping across contextual signals on Visuelle 2.0 and TBH Global datasets.

Mapping	Contextual Signal	Visuelle 2.0			TBH Global			Avg. Rank
		M ↓	S ↓	W ↓	M ↓	S ↓	W ↓	
Max Norm	Trends	19.937	0.599	1.189	36.332	0.513	1.101	5.1
	POP	20.677	0.600	1.233	35.935	0.510	1.084	5.3
	Ref. Sales	20.059	0.597	1.196	35.921	0.510	1.083	4.3
Fourier Mapping	Trends	*15.551*	*0.567*	*0.927*	**29.028**	*0.449*	**0.772**	1.6
	POP	15.668	0.568	0.934	29.178	0.458	0.779	3.0
	Ref. Sales	**15.246**	**0.564**	**0.909**	*29.089*	**0.445**	**0.772**	**1.3**

Note. Best results are bolded, and second-best results are italicized.

The results clearly show that Fourier Mapping consistently outperforms Max Normalization regardless of the contextual signal used. In particular, applying Fourier Mapping to reference item sales achieves the best performance across all metrics and datasets.

These findings support our claim that Fourier Mapping is distribution-agnostic—it does not rely on absolute magnitude ranges, unlike Max Normalization—and better captures fine-grained variations in contextual signals. The strong gains observed when combining Fourier Mapping with reference item sales also corroborate the findings from our earlier analysis of frequency sensitivity.

#### Ablation study on Fourier frequency scale.

Fourier Mapping transforms individual sales values into frequency-based embeddings, allowing the model to learn both low-frequency and high-frequency patterns effectively. However, the frequency scaling factor *B*, which determines the range of frequencies used in the transformation, plays a crucial role in controlling the balance between long-term trends and short-term variations. A larger *B* value concentrates the embedding on lower-frequency components, a smaller *B* introduces higher-frequency components, making the model more sensitive to short-term variations. A suboptimal *B* value may lead to excessive smoothing (underfitting) or over-sensitivity to noise (overfitting).

For all previous experiments, we set the frequency scaling factor to *B* = 10000 as the default value, based on preliminary tuning and prior research on spectral bias in neural networks. To further analyze the impact of this parameter, we conduct an ablation study by varying *B* across multiple values:


$$\begin{array}{c} B\in \{2500,5000,10000,20000,40000\} \end{array}$$ (24)

### Experimental results

As shown in Table 6, the choice of the frequency scaling parameter *B* in Fourier Mapping has a clear impact on forecasting performance. Among the tested values, the setting *B* = 5000 achieved the best performance across all evaluation metrics indicating a well-balanced trade-off between sensitivity and robustness. In comparison, the default value *B* = 10000 yielded slightly worse results. These findings suggest that moderate frequency scales (e.g., *B* = 5000) may offer better expressiveness for Fourier embeddings.

**Table 6:** Effect of frequency scaling (*B*) in Fourier Mapping on forecasting performance.

Frequency Scaling (*B*)	M ↓	S ↓	W ↓
2500	15.237	0.571	0.908
5000	**15.111**	**0.563**	**0.901**
10000	15.246	0.564	0.909
20000	15.439	0.572	0.920
40000	15.508	0.572	0.924

Although *B* is currently treated as a fixed hyperparameter, these results suggest that its value significantly affects model performance. Therefore, future work may explore strategies to learn or adaptively tune *B* from data, potentially enabling the model to dynamically adjust its frequency sensitivity.

### Category-group-wise sensitivity analysis

To examine whether the optimal value of *B* depends on broader product characteristics, we grouped the 27 categories in Visuelle 2.0 into four category groups and evaluated EMP across the same range of frequency scales. The category groups and their constituent items are summarized above. The corresponding results are presented in Table 7.

- **Top**: long sleeve, short sleeves, sleeveless, solid color top, patterned top, drop sleeve, printed shirt
- **Skirt**: culottes, miniskirt, gitana skirt, capris, maxi
- **Dress**: doll dress, long dress, trapeze dress, shirt dress, sheath dress, kimono dress
- **Coat and Other**: short coat, medium coat, long coat, long duster, medium cardigan, short cardigan, long cardigan, jumpsuit, shorts

**Table 7:** Category-group-wise analysis of frequency scaling parameter *B* on Visuelle 2.0.

Category Group	B	M ↓	S ↓	W ↓
Top	2500	3.766	0.555	1.114
	5000	3.711	0.555	1.112
	10000	**3.664**	**0.555**	**1.112**
	20000	4.088	0.569	1.240
	40000	3.729	0.558	1.131
Skirt	2500	**5.075**	**0.678**	**2.254**
	5000	5.714	0.693	2.538
	10000	6.323	0.708	2.809
	20000	7.656	0.730	3.400
	40000	8.952	0.744	3.976
Dress	2500	**3.011**	**0.601**	**1.304**
	5000	3.033	0.602	1.314
	10000	3.116	0.605	1.350
	20000	3.408	0.616	1.477
	40000	3.748	0.628	1.624
Coat and Other	2500	**3.182**	**0.625**	**1.852**
	5000	3.809	0.648	2.217
	10000	5.265	0.683	3.065
	20000	6.043	0.700	3.517
	40000	7.497	0.722	4.364

This category-group-wise analysis shows that the optimal frequency scaling varies meaningfully across product groups. For the Top group, the best performance is achieved at *B* = 10000, suggesting that these relatively stable categories benefit from lower-frequency embeddings. In contrast, the Skirt, Dress, and Coat and Other groups achieve their lowest errors at *B* = 2500, indicating that these item types gain more from higher-frequency sensitivity. These findings suggest that the optimal frequency scaling is category-group-dependent, which in turn suggests that future work could explore mechanisms for learning or adjusting *B* according to category-group characteristics.

### Computational efficiency analysis

To further examine the practical feasibility of the proposed framework, we analyze the computational efficiency of EMP in terms of (i) single-sample inference time and (ii) the computational cost of inverse Fourier mapping, both of which are important for understanding the practical deployability of EMP in real-world retail environments.

### Single-sample inference time

As shown in Table 8, EMP operates solely on internal signals and therefore performs inference without any external API calls. In contrast, all baseline models except Embedding KNN depend on external sources: Attribute KNN, Attr+Emb KNN, Image RNN, Multi-Modal RNN, Cross-Attention RNN, and GTM-Transformer all require external trends API, while POP instead relies on Google Image Search followed by crawler-based image download. For these baselines, we report end-to-end inference time including both external signal retrieval and model forward computation, reflecting the total delay that would be observed in real deployment settings.

**Table 8:** Single-sample end-to-end inference time.

Model	External Signal Required	Inference Time (sec)
Attribute KNN	Yes (trends API)	3.31
Embedding KNN	No	0.28
Attr+Emb KNN	Yes (trends API)	3.49
Image RNN	Yes (trends API)	3.70
Multi-Modal RNN	Yes (trends API)	4.37
Cross-Attention RNN	Yes (trends API)	4.66
GTM-Transformer	Yes (trends API)	4.80
POP	Yes (image crawler)	21979
EMP (Proposed)	No	4.45

EMP’s total inference time (4.45 seconds) consists of two internal components: the reference item retrieval stage (Stage 1: 2.81 seconds) and the forecasting stage (Stage 2: 1.64 seconds). Although this overall duration is comparable to the end-to-end inference time of several baselines, the operational flow differs. Baseline models rely on external signals that must be obtained from trend-query services (or image crawler in the case of POP) before their forecasting modules can run, whereas EMP performs its entire inference process using internal inputs only. This removes the need for any external data preparation during inference while still achieving substantially higher forecasting accuracy.

### Computational cost of inverse Fourier mapping

The inverse Fourier mapping step reconstructs scalar sales values by comparing the predicted Fourier embedding against a precomputed basis {γ(0),γ(1),…,γ(M−1)}, where *M* denotes the size of the discretized sales range. Computing inner products between the predicted embedding and all *M* basis vectors results in a theoretical complexity of 𝒪(M·d), with *d* fixed. To examine whether this cost becomes problematic when the sales range grows large—a central concern for real-world deployment—we synthetically expand the basis size by factors of 2×, 4×, 10×, 100×, 1000×, and 10000×, and measure the runtime of Stage 2, which includes both the decoder forward computation and the inverse mapping.

As shown in Table 9, the runtime of Stage 2 remains essentially constant even when the Fourier basis is expanded by several orders of magnitude, with only a minor increase at the 10000× setting. This indicates that the inverse mapping is not a meaningful source of computational overhead, as the Transformer decoder dominates inference cost. Moreover, the inverse mapping can be computed efficiently through a matrix multiplication followed by an argmax operation. These results show that the inverse Fourier Mapping scales well and does not introduce a computational bottleneck in our integer valued forecasting framework.

**Table 9:** Runtime of Stage 2 under increasing Fourier basis sizes.

Basis Expansion	Stage 2 Runtime (sec)
1× (default)	1.64
2×	1.63
4×	1.56
10×	1.62
100×	1.61
1000×	1.62
10000×	2.04

## Discussion

### Comparison of Lipschitz Constants in Fourier Mapping and Max normalization

Prior research [37–39] has demonstrated that Lipschitz continuity is a crucial property in deep learning models, as it governs how sensitive the output embeddings are to changes in the input. A higher Lipschitz constant indicates that small input changes cause larger variations in the embedding space, which is beneficial for capturing high-frequency patterns. However, an excessively high Lipschitz constant may lead to instability and overfitting, whereas a very low Lipschitz constant can cause underfitting [38].

This section empirically compares the Lipschitz constants of Fourier Mapping and Max Normalization to assess their impact on sales forecasting.

### Lipschitz constant calculation

The Lipschitz constant is defined as:


$$\begin{array}{c} L=\frac{\left\|\gamma (s_{1})-\gamma (s_{2})\right\|}{\left|s_{1}-s_{2}\right|} \end{array}$$ (25)

where γ(s) represents the transformation applied to the scalar sales value *s*.

### Max normalization

Max Normalization scales the input by the maximum observed value:


$$\begin{array}{c} s^{\prime }=\frac{s}{\max(x)} \end{array}$$ (26)

Thus, its Lipschitz constant is:


$$\begin{array}{c} L_{\text{max\_norm}}=\frac{1}{\max(x)} \end{array}$$ (27)

For our dataset, where max(x)=751, the computed Lipschitz constant is:


$$\begin{array}{c} L_{\text{max\_norm}}=\frac{1}{751}\approx 0.001332 \end{array}$$ (28)

### Fourier mapping

Fourier Mapping transforms scalar sales values into sinusoidal embeddings. As derived in Eq (20), the Euclidean distance between two transformed values leads to the following Lipschitz constant:


$$\begin{array}{c} L_{\text{fourier}}=\frac{\sqrt{\sum_{k=1}^{d/2}2-2\cos(f_{k}(s_{1}-s_{2}))}}{\left|s_{1}-s_{2}\right|} \end{array}$$ (29)

we compute the Lipschitz constant over all $s_{1}-s_{2}$:


$$\begin{array}{c} L_{\text{fourier}}\in [0.0264,3.7142] \end{array}$$ (30)

which is always greater than Max Normalization’s Lipschitz constant of 0.001332.

### Impact of Fourier Mapping on high-frequency sensitivity

Our empirical analysis confirms that Fourier Mapping consistently exhibits a higher Lipschitz constant than Max Normalization across all values of $\left|s_{1}-s_{2}\right|$. Specifically, the Lipschitz constant of Fourier Mapping ranges from 0.0078 to 0.1061, with values that consistently exceed the Lipschitz constant of Max Normalization, which is 0.001332.

This property indicates that Fourier Mapping is significantly more sensitive to small numerical changes in sales values, enabling it to preserve fine-grained variations that are critical for accurate demand forecasting. In contrast, Max Normalization flattens small-scale differences, potentially suppressing high-frequency components and degrading the model’s ability to detect short-term dynamics.

These findings align with prior research on spectral bias, which suggests that neural networks inherently struggle to learn high-frequency patterns unless such information is explicitly preserved in the input representation [28]. By amplifying high-frequency sensitivity through a larger Lipschitz constant, Fourier Mapping mitigates spectral bias and enhances the model’s capacity to capture subtle, short-term fluctuations in sales data.

### Frequency sensitivity of contextual signals

To justify our choice of applying Fourier Mapping specifically to reference item sales, we analyze the frequency sensitivity of contextual scalar signals used in the model, including Google Trends and POP signals. The goal is to determine which signals exhibit stronger high-frequency components and thus benefit more from frequency-aware embeddings.

### Fourier embedding and frequency indexing

Each signal is transformed using Fourier Mapping, which embeds scalar values into a high-dimensional sinusoidal representation as previously defined in Eq (2). The frequency components *f*_*k*_ follow the exponential decay formulation described in the same section.

Earlier frequency indices (i.e., lower values of *k*) correspond to higher frequency components, while later indices correspond to lower frequencies. Thus, in the embedding vector, dimensions near the beginning (e.g., indices 0–3) represent high-frequency patterns, and those near the end (e.g., indices 510–511) represent low-frequency patterns.

### Spectral contribution analysis via PCA

We flatten each Fourier-embedded signal across time and apply Principal Component Analysis (PCA) to extract dominant spectral patterns. Let $X\in {\mathbb{R}}^{N\times d}$ be the matrix of Fourier-embedded sequences, centered by subtracting the mean vector μ. The sample covariance matrix is defined as:


$$\begin{array}{c} C=\frac{1}{N}(X-1\mu^{⊤}{)}^{⊤}(X-1\mu^{⊤}), \end{array}$$ (31)

and the first principal component vector ${𝐮}_{1}\in {\mathbb{R}}^{d}$ is given by:


$$\begin{array}{c} C{𝐮}_{1}=\lambda_{1}{𝐮}_{1}, \end{array}$$ (32)

where $\lambda_{1}$ is the largest eigenvalue of *C*. Each entry *u*_1,*j*_ represents the loading of the *j*-th input dimension (e.g., a sinusoidal frequency component) in the first principal direction.

We then compute the average magnitude contribution of each frequency pair (sin,cos) as:


$$\begin{array}{c} {\bar{w}}_{i}=\frac{\left|u_{1,2i}|+|u_{1,2i+1}\right|}{2},\;i\in \{0,1,\ldots ,255\}, \end{array}$$ (33)

and define the expected frequency index as:


$$\begin{array}{c} 𝔼[f]=\frac{\sum_{i=0}^{255}i\cdot {\bar{w}}_{i}}{\sum_{i=0}^{255}{\bar{w}}_{i}}. \end{array}$$ (34)

### Results and interpretation

The computed expected frequency indices are as follows:

- Google Trends – Category: 81.16
- Google Trends – Fabric: 67.03
- Google Trends – Color: 58.37
- POP signals: 81.05
- Reference Item Sales: **54.45**

Lower expected frequency indices indicate stronger presence of high-frequency components. Among all contextual signals, reference item sales exhibit the highest high-frequency sensitivity. This result shows that these signals contain fine-grained temporal variations—sharp changes, local trends, and short-term fluctuations—that are critical for accurate sales forecasting.

Applying Fourier Mapping to such high-frequency-rich signals is particularly effective, as it preserves these variations in the embedding space. In contrast, signals like POP signals tend to reflect low-frequency information and thus do not benefit as much from frequency-aware transformation. This analysis supports our design decision to apply Fourier Mapping selectively to reference item sales and not to external signals.

### Impact of discretized inverse mapping on prediction accuracy

The proposed model predicts continuous Fourier embeddings, which must be post-processed into scalar sales values via Inverse Fourier Mapping. This operation, defined in Eq. 17, identifies the most similar discrete basis vector using dot-product similarity against a precomputed set of Fourier embeddings.

Because this inverse mapping step projects from a continuous embedding space to a discrete output domain, small deviations in the predicted embeddings could, in principle, lead to incorrect sales values being selected. In other words, quantization error may occur if the predicted Fourier embedding does not exactly match any of the precomputed basis vectors, causing the argmax operation to round to a neighboring sales value.

To understand the extent to which such discretization affects prediction accuracy, we conducted a controlled robustness analysis. Specifically, we injected Gaussian noise into ground-truth Fourier embeddings to emulate the kind of perturbation introduced by real-world prediction errors. This approach allows us to isolate the quantization effect, independent of the model’s architecture or optimization.

**Fig 4:**
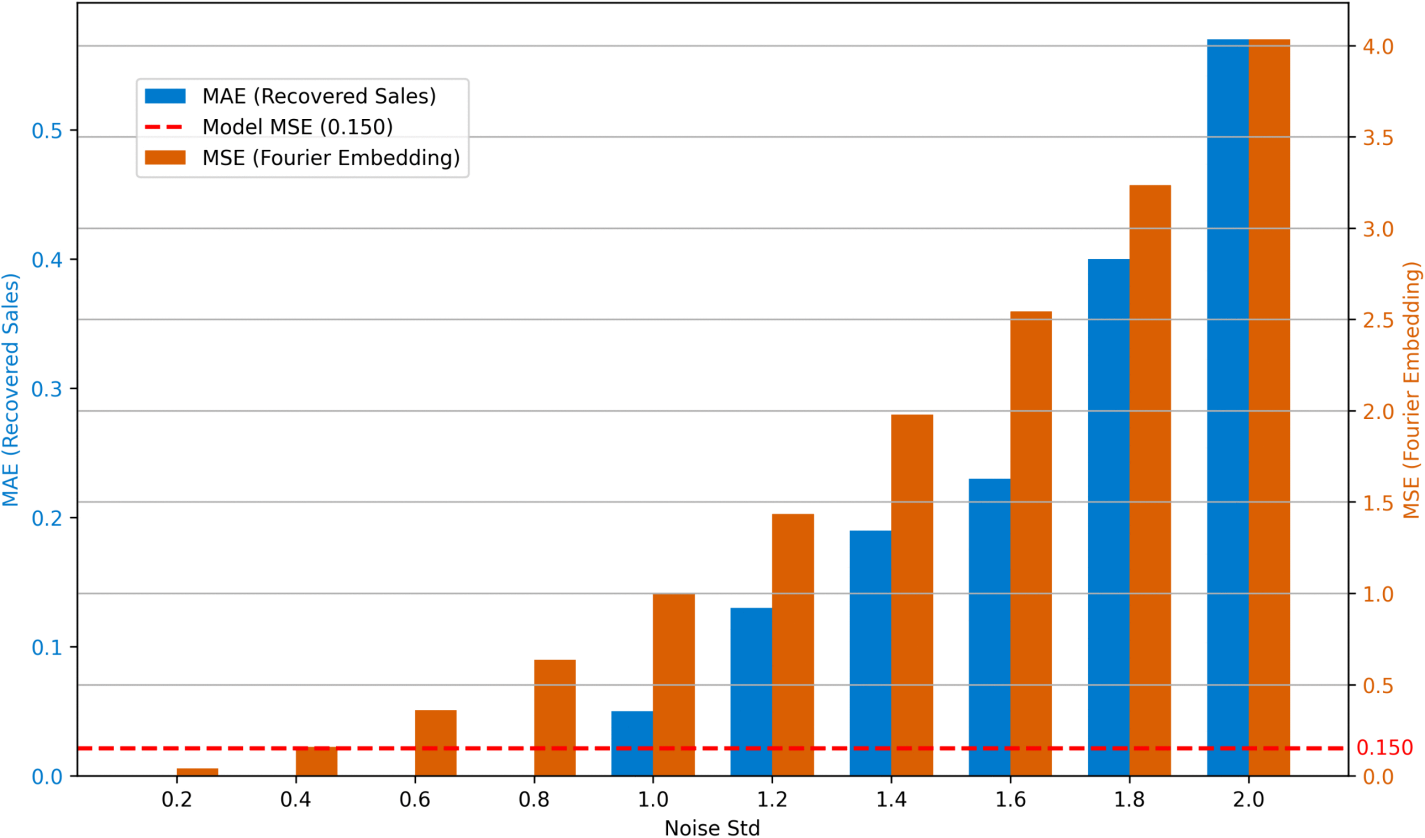
Effect of embedding noise on inverse mapping accuracy. Note. Left axis: MAE in recovered scalar sales. Right axis: MSE in Fourier embedding space. The red dotted line marks the model’s average embedding MSE.

We sampled noise from multiple standard deviations σ∈0.2,0.4,…,2.0 and, for each level, measured the embedding-level Mean Squared Error (MSE) and the Mean Absolute Error (MAE) of the resulting recovered sales values. As shown in Fig 4, the inverse mapping procedure remained stable even under substantial noise. Notably, the average MAE stayed below 0.1 for σ≤1.0, and was exactly 0.0 for σ=0.4. This suggests that small perturbations in Fourier embeddings do not cause erroneous discretization.

Given that the trained EMP model exhibits an average embedding-level MSE of approximately 0.150, this corresponds to a noise level close to σ=0.4 in our controlled setting. Therefore, we conclude that quantization error introduced by the discretized inverse mapping remains negligible under realistic inference conditions, and does not materially affect final predictive accuracy.

### Interpretation of Fourier mapping behavior

A deeper examination of Fourier Mapping reveals several geometric behaviors that help explain its strong performance within the EMP framework. As illustrated in S1 Fig, Fourier Mapping transforms scalar sales values into frequency based embeddings whose structure preserves meaningful numerical relationships.

### Empirical analysis of Euclidean distance in Fourier space

One of the defining characteristics of Fourier Mapping is how it encodes numerical differences in an interpretable way. Unlike traditional normalization techniques, Fourier Mapping ensures that numerical differences are reflected in the embedding space in a way that captures both local and global relationships.

Key observations from S1 Fig include:

- The Euclidean distance is lowest when the sales value equals the ground truth.
- The distance increases non-linearly as values deviate, unlike uniform scaling.
- Embeddings retain meaningful numerical relationships suited for MSE-based regression.

### Empirical analysis of similarity trends in Fourier embeddings

Another key property of Fourier Mapping is its ability to maintain smooth similarity transitions across different sales values. As shown in S1 Fig, this behavior exhibits:

- Similarity is highest near the ground truth.
- Similarity decays gradually and periodically with increasing difference.
- This suggests Fourier Mapping captures both global structure and local variance.

## Limitations

### Multimodal redundancy and computational efficiency

A potential limitation of the proposed framework lies in the presence of redundant information across different modalities. In multimodal settings, certain attributes—such as categorical or semantic information—may be simultaneously encoded in multiple sources, including textual descriptions and structured metadata. This overlap can lead to redundant representations within the fused feature space.

Such redundancy introduces potential computational inefficiency, as the model processes multiple features that convey similar information without explicitly filtering them. In the current design, all modality-specific features are integrated through a fusion network without an explicit mechanism for feature selection or redundancy reduction.

To address this issue, future work could explore more efficient feature selection strategies, such as learnable gating mechanisms that explicitly regulate the contribution of each modality.

## Conclusion

This paper introduced EMP (Enhanced Multi-modal Prediction), a framework for zero-shot time series forecasting of fashion sales. EMP captures inter-item relationships, effectively models high-frequency variations in sales values, improves forecasting accuracy, and eliminates the need for dataset-specific scaling. The scale-aware representation of Fourier Mapping allows pre-trained time series foundation models to operate without dataset-specific normalization. However, whether this property consistently leads to improved performance in foundation model settings remains an open question, which we leave for future work.

Experiments on TBH Global and Visuelle 2.0 datasets demonstrate that EMP outperforms state-of-the-art (SOTA) models. Lipschitz constant analysis shows that Fourier Mapping captures high-frequency sales variations more effectively than conventional scaling. Given that reference item sales contain rich high-frequency signals, selectively applying Fourier Mapping to these internal signals helps preserve fine-grained temporal patterns, contributing to EMP’s improved predictive performance.

Finally, we acknowledge that one of the two datasets used in our experiments—TBH Global—is not publicly available. While we have adhered to the journal’s policy on data availability, we recognize that this may limit reproducibility for some readers. To address this, we emphasize EMP’s strong and consistent performance on the publicly available Visuelle 2.0 dataset, which serves as a transparent benchmark for future research.

## Supporting information

S1 Fig Experimental Fourier Embedding Analysis. Note. Left: Euclidean Distance (MSE Loss) from the ground truth (0, 350, 700). Right: Dot Product Similarity with respect to the ground truth. The structured distance and similarity trends demonstrate Fourier Mapping’s ability to preserve numerical relationships while introducing periodicity.
(TIF)

S2 Fig Comparison of model predictions on Visuelle 2.0 Dataset. Note. In all but the first plot, GTM-Transformer predicted significantly higher values, expanding the y-axis and reducing visible differences among models. Out-of-range values are marked separately as “GTM (Out of range).”
(TIF)
